# Prox1 Inhibits Proliferation and Is Required for Differentiation of the Oligodendrocyte Cell Lineage in the Mouse

**DOI:** 10.1371/journal.pone.0145334

**Published:** 2015-12-28

**Authors:** Kentaro Kato, Daijiro Konno, Martin Berry, Fumio Matsuzaki, Ann Logan, Alicia Hidalgo

**Affiliations:** 1 NeuroDevelopment Group, School of Biosciences, University of Birmingham, Edgbaston, Birmingham, United Kingdom; 2 Institute of Inflammation and Ageing, College of Medical and Dental Sciences, University of Birmingham, Edgbaston, Birmingham, United Kingdom; 3 Laboratory for Cell Asymmetry, RIKEN Center for Developmental Biology, 2-2-3 Minatojima Minamimachi, Chuo-ku, Kobe, Japan; Instituto Cajal-CSIC, SPAIN

## Abstract

Central nervous system injury induces a regenerative response in ensheathing glial cells comprising cell proliferation, spontaneous axonal remyelination, and limited functional recovery, but the molecular mechanisms are not fully understood. In *Drosophila*, this involves the genes *prospero* and *Notch* controlling the balance between glial proliferation and differentiation, and manipulating their levels in glia can switch the response to injury from prevention to promotion of repair. In the mouse, Notch1 maintains NG2 oligodendrocyte progenitor cells (OPCs) in a progenitor state, but what factor may enable oligodendrocyte (OL) differentiation and functional remyelination is not understood. Here, we asked whether the mammalian homologue of *prospero*, *Prox1*, is involved. Our data show that Prox1 is distributed in NG2+ OPCs and in OLs in primary cultured cells, and in the mouse spinal cord *in vivo*. siRNA *prox1* knockdown in primary OPCs increased cell proliferation, increased NG2+ OPC cell number and decreased CC1+ OL number. *Prox1* conditional knockout in the OL cell lineage in mice increased NG2+ OPC cell number, and decreased CC1+ OL number. Lysolecithin-induced demyelination injury caused a reduction in CC1+ OLs in homozygous *Prox1-/-* conditional knockout mice compared to controls. Remarkably, *Prox1-/-* conditional knockout mice had smaller lesions than controls. Altogether, these data show that Prox1 is required to inhibit OPC proliferation and for OL differentiation, and could be a relevant component of the regenerative glial response. Therapeutic uses of glia and stem cells to promote regeneration and repair after central nervous system injury would benefit from manipulating Prox1.

## Introduction

Glial cells proliferate throughout life in response to neuronal activity, conveying homeostatic regulation of structure and function. NG2+ Oligodendrocyte Progenitor Cells (OPCs) proliferate and differentiate to produce oligodendrocytes (OLs), which ensheath and myelinate axons, provide trophic factors that maintain neuronal survival, regulate ion homeostasis and enable saltatory conduction in the central nervous system (CNS) [1–5]. Disregulation of OPC and OL number leads to gliomas and demyelinating diseases, like Multiple Sclerosis. CNS damage and acute OL loss induce a robust regenerative response that promotes OPC proliferation, OL differentiation and spontaneous remyelination [2,6,7]. This, however, does not culminate in full functional repair as the lesion is invaded by microglia, macrophages and astrocytes that form the glial scar, inhibit axonal growth, cause myelin breakdown and cell death [8,9]. Transplantation of glial cells to spinal cord injury lesions results in limited yet remarkable recovery of locomotion in mammals, including humans [10]. Thus, uncovering the molecular mechanisms that control NG2+ OPC proliferation and their differentiation into OLs is essential to understand CNS structural plasticity, the endogenous glial regenerative response to injury, and how to enhance repair [2].

*Notch1* is expressed in OPCs during development and in the adult, and it inhibits OL differentiation maintaining OPCs in a progenitor state in culture and in vivo [11,12]. *Notch1* conditional-knock-out (CKO) in OPCs in mice induces OL differentiation [12], indicating that Notch1 antagonises a factor that promotes OL differentiation. Yet, the involvement of Notch1 in the glial response to injury in the mouse is unresolved. Upon injury, *Notch1* expression increases in OPCs, correlating with OPC proliferation at the lesion boundaries, and with remyelination in mice [13,14]. However, *Notch1-CKO* targeted to OPCs and OLs did not affect the regenerative response to Cuprizone-induced or experimental autoimmune encephalomyelitis (EAE) demyelination in mice [13,15]. Nevertheless, the consensus is that injury induces the proliferation of Notch1+ NG2+ OPCs in mammals, but it is unknown what factor may antagonise Notch1 to drive OL differentiation conducive to re-myelination.

*Drosophila* is a powerful model organism to identify gene networks and function. The glial regenerative response of neuropile-associated glia to CNS injury in fruit-flies requires the antagonistic functions of the *Notch1* homologue, *Notch*, and *prospero (pros)* [16,17]. Pros inhibits glial proliferation and promotes differentiation, including morphology, axonal enwrapment, and expression of glial differentiation markers such as Ebony and Glutamine Synthetase 2 involved in neurotransmitter recycling. Notch inhibits glial differentiation and promotes proliferation in flies. Nevertheless, glial proliferation in development and upon injury requires both Pros and Notch, as although they have opposite effects on glia, they maintain each other’s expression, enabling differentiated glia to retain mitotic potential. This feedback loop between Notch and Pros provides a homeostatic mechanism to regulate glial number in development and upon injury [17].

Whether mammalian OL lineage cells express the *pros* homologue, *Prox1*, and might influence the glial regenerative response to spinal cord injury, is unknown. Tentative evidence suggests Prox1 could be involved. Prox1 promotes cell cycle exit and induces differentiation in many contexts in mammals [18]. In the retina, Prox1 antagonises Notch1 function in the generation of new neurons [19]. Prox1 was observed in OL lineage cells in the cortex of the mouse[20]. Clonal analyses in mouse brains suggest that glioma originate from NG2+ OPCs, and glioma cells are known to express *Prox1* [21,22]. Thus, it was compelling to test the involvement of Prox1 in the mammalian OL cell lineage. Here, we investigate the function of Prox1 in the OL cell lineage, and in the glial regenerative response to demyelination in the adult mouse spinal cord.

## Materials and Methods

### Animals

Depending on the experiments, animal procedures were licensed by the UK Home Office and approved by the University of Birmingham's Biomedical Ethics Review Sub-Committee, or reviewed and approved by the RIKEN Center for Developmental Biology, Japan. C57/BL6 mouse were used for section preparation of spinal cords, and for OPC primary culture. Prox1-CKO experiments were carried out using the *Olig2-CreER* mouse line, whereby the *Olig2* promoter drives expression of *CRE-recombinase* only in the OL cell lineage [23,24]. *Olig2CreER KI* mice [24] and *Prox1 F/F* mice were used [23]. In progeny mice from the two lines above, Tamoxifen application induces the nuclear localisation of CreER Recombinase, leading to the flip-out only in OPCs and OLs of the *Prox1* cDNA, which had been inserted downstream of the 5’UTR, in the first exon of the *Prox1* gene. This resulted in the knock-out of the *Prox1* coding region and the expression of GFP under the control of the *Prox1* promoter in the OL cell lineage. *Prox1 F/+;Olig2-CreER KI/+* and *Prox1 F/F* mice were crossed to obtain experimental *Prox1 F/F;Olig2-CreER KI/+* mice and control *Prox1 F/+;Olig2-CreER KI/+* mice. Tamoxifen was applied to induce *Prox1* flip-out at week 5 after birth, and the spinal cords were harvested 5 weeks later. Genotyping was performed by PCR analysis with specific primers for the *Olig2CreER* allele (5’-TCGAGAGCTTAGATCATCC-3’, 5’-AGCATTGCTGTCACTTGT-3’, 5’-CACCGCCGCCCAGTTTGTCC-3’) and *Prox1-CKO* allele (5’-CAGCCCTTTTGTTCTGTTGGCC-3’, 5’-CAGATGCTGTCCCTACCGTCC-3’).

We quantified the GFP+ cells, and found that whereas in *Prox1-CKO+/-* heterozygotes virtually all GFP+ cells were Prox1+ (average 97% n = 6 mice), there was only a 30% reduction in the percentage of Prox1+ cells amongst the GFP+ cells in *Prox1-CKO-/-* homozygous mice (average 68%, n = 7 mice). As GFP is only detectable in the OL cell lineage if a knockout event takes place, this would imply that Prox1 protein persists presumably due to its slow turnover. The frequency of knock-out events also appears to have been rather low: only 4% of OL cell lineage cells in the ventral funiculus of *Prox1-CKO+/-* heterozygous mice were GFP+, and only 3% were GFP+ in homozygotes (n = 6 and 7 mice respectively). This might have been due to low Tamoxifen application.

### Tamoxifen administration

To generate *Prox1-CKO* heterozygous/homozygous cells, Tamoxifen (3mg/mouse, Sigma), dissolved in peanut oil (Sigma) at a final concentration of 10 mg/ml, was applied by gavage to *Prox1 F/F;Olig2-CreER Kl/+* mice and control *Prox1 F/+;Olig2-CreER Kl/+* mice twice at week 4. The spinal cords as intact samples were harvested at week 11, which received intraperitoneal injection of 5mg/ml BrdU PBS solution (50mg/kg), 3 times a day with 2 hours interval, for four days at 3 weeks before fixation. 3 mice (2 female, 1 male) for heterozygotes and 4 mice (2 female, 2 male) for homozygotes were sacrificed.

### LPC induced demyelination

It has been previously shown that DNA synthesis occurs in OPCs approximately 3 days after injury, and OL differentiation and remyelination occur by day 14 after LPC injection [25,26]. Thus, we applied Tamoxifen at week 5 after birth to induce the *Prox1-CKO* event, we injected LPC or PBS (as a control) into the ventral funiculus of the spinal cords at week 8, applied BrdU 3 days later, and harvested the spinal cords at day 14 post-LPC-injection (week 10).

PBS injections in heterozygous (5 mice: 2 males, 3 females) and in homozygous mice (5 mice: 2 males, 3 females), and LPC injections in heterozygous (5 mice: 3males, 2 females) and in homozygous mice (6 mice: 3 males, 3 females) were carried out. The mice were anaesthetised with inhaled isoflurane/oxygen, supplemented with buprenorphine. Dorsal laminectomies were performed at the level of T8/T9 vertebra. After the dura mater was incised transversely, 2μl of PBS (as a control) or 1% L-a-lysolecithin (Lysophosphatidylcholine; Sigma) PBS solution were slowly delivered into the ventral funiculus by a grass capillary attached to a syringe. After 2 days of injection, mice received intraperitoneal injection of 5mg/ml BrdU PBS solution (50mg/kg); 3 times a day with 2 hours interval for 2 days. The mice were killed 14 days after the PBS or LPC injection, and the spinal cords were harvested.

### Antibodies

Antibodies used in this study were: rabbit anti-NG2 (1:400, Millipore), mouse anti-CC1 (CC-1, 1:400, MERC), sheep anti-BrdU (1:400, Exalpha Biologicals), mouse anti-MBP (1:4000, Covance), goat anti-Notch1ICD (1:50, Santa Cruz), goat anti-Prox1 (1:50, R & D system), rat anti-PDGFRα (1:100, eBioscience), rat F4/80 (1:1000, Serotec), chick anti-GFP (1:2000, Aves), mouse anti-GFP (1:400, Life Technologies), rabbit anti-GFP (1:500, Life Technologies), Alexa 488, 594, 647 conjugated donkey secondary antibodies (1:400, Life Technologies), biotinylated donkey anti-goat and biotinylated donkey anti-chicken (1:400, Life Technologies), and Streptavidin 488, 546 (1:400, Life Technologies).

### Tissue preparation and immunostaining

Mice were killed by anaesthetic overdose, and perfusion fixed with 4% paraformaldehyde (TAAB Laboratories). Subsequently, the spinal cords were dissected, fixed and cryoprotected with sucrose. Then, they were embedded in OCT (Miles Inc.), and frozen with dry ice. Samples were sectioned horizontally 15 μm thick at -20°C (Bright Instrument), collected on Vectabond coated slides (Vector laboratories), air dried, and maintained at -20°C. For immunostaining, the sections and cells on coverslips from cell culture were washed with PBS, permeabilised with 0.3% Triton X-100 (Sigma), and blocked with 5% normal donkey serum (Sigma) or normal goat serum (Vector laboratories). Sections were also blocked with donkey anti-mouse IgG Fc (1:100, Jackson Immunoresearch) when the mouse derived primary antibodies were used. Incubation with primary antibodies was performed at 4°C overnight, and with fluorescent-conjugated secondary antibodies was for 30 minutes at room temperature (RT). Nuclei were stained with DAPI (Sigma), and samples were mounted with 50% Glycerol. To detect BrdU, the sections/cells were treated with 2M HCl for 20 minutes at RT after immunolabelling for other proteins and fixation. To detect MBP, the sections were treated with 95% EtOH 5% Acetic Acid at RT for 15 min after immunolabelling for other proteins and fixation. After washing and blocking, the sections were treated with anti-MBP antibodies conjugated with Alexa fluor 546 (Zenon Mouse IgG1 labelling kit, Life technologies), for 15 minutes at RT. Confocal microscopy was done with Leica SP2-AOBS confocal microscope. Obtained images were analysed with ImageJ and processed with Photoshop (Adobe).

### Cell culture

Mouse OPCs were purified from P0-P2 C57/BL6 mouse brains by the shaking method and we achieved between 61 and 87% cell purity as in the original protocol [27,28]. OPCs were plated at a density of 20,000 cells per 9 mm round Poly-ornithine coated coverslips (Sigma). They were maintained in NBM OPC medium [28] with a slight modification; NBM (Life Technologies) supplemented with B27 (Life Technologies), 4mM L-Glutamine (Sigma), 1mM Sodium Pyruvate (Sigma) and 10 ng/ml PDGF-AA (Peprotech). As a differentiation medium, NBM supplemented with B27, 4mM L-Glutamine, Sodium Pyruvate, 10 ng/ml CNTF (Peprotech) and 30 ng/ml T3 (Sigma) was used.

### siRNA-mediated Gene silencing

Primary OPCs were transfected with *Prox1-siRNA* and *Notch1-siRNA*, and a day later were shifted to a differentiation-inducing medium where they were maintained for 72 hours. On-TARGET plus siRNA SMARTpools (Thermo Fisher Scientific) against mouse Prox1 (L-058437-01-0005, UGGAGAAGUAUGCGCGUCA, UAGCACAGGCUCCGAAGUA, AGUCGAACGUACUCCGCAA, GAACAAGCCUAAGCGAGAA) and Notch1 (L-041110-00-0005, GCCCGUGGAUUCAUCUGUA, AGACAGCUAUGCUACUUAU, GAGCGUAUGCACCACGAUA, CAAGAUUGAUGGCUACGAA) were transfected to OPCs using Ribocellin siRNA Transfection Reagent (BiocellChallenge) at day-2 of primary culture. The medium containing siRNA was replaced with differentiation medium at day-3. Subsequently, the medium was replaced with differentiation medium containing 10μM BrdU (Sigma) at day-4, then the cells were fixed at day-5. The efficiency of knockdown of Prox1 was determined in three independent experiments by Immunostaining, followed by confocal microscopy with 40x lens and 4x zoom on Leica SP6 confocal microscope.

### Quantification and statistical analysis

Images stained by immunofluorescence were acquired using a Leica SP2-AOBS confocal microscope. Image processing—thresholding, and measurement of area size and automatic (ITCN plug-in)/manual counting of cells (cell counter plug-in)—were done using ImageJ. For the cell count analysis on cell culture, 3 coverslips with primary OPCs were prepared from each of three to four independent experiments. After immunostaining, more than 100 of cells per coverslip were scanned using a confocal microscope with a 40x lens. The effects of gene knockdown in OPC primary culture were examined by counting the number of NG2+, CC1+ and BrdU+. This was done manually using the cell counter plug-in and setting the expression with threshold. For the analysis of spinal cords, we focused on the ventral funiculus. Images were obtained with a 20x lens, and stitched using Fiji software to cover the entire width and length of demyelinated area or equivalent area in intact spinal cords. For cell number, the counts were made on two sections per animal when possible. The numbers of Prox1 and DAPI were counted with the ITCN plug-in. The counts of CC1+, GFP+, NG2+ and BrdU+ were done manually, and only when they colocallised with DAPI (nuclear). For the count of cells in the demyelinated area, a region of interest (ROI) was set to a 100μm wide band from the limits of nuclear-dense area (close approximate to the demyelinated area). The NG2-positive pixels were measured within this ROI instead of cell number because of difficulties of identifying cell bodies. For the LPC treatment experiments, the MBP-negative area was measured from laser scanning confocal microscopy images stained with anti-MBP, by drawing the outline of the lesion in ImageJ. Lesion volume was estimated from bright field images of all the available spinal cord sections (i.e. using also sections that had not been stained with antibodies), taken using a Leica MZFLIII dissecting microscope. The lesions were identified visually from the background white matter, and they were comparable in shape and size to the MBP-negative areas in the stained sections of each spinal cord. The lesion area was first measured by drawing the outline using ImageJ, and the volume in each section was obtained by multiplying area by section thickness, 15μm. The area in missing sections was extrapolated from adjacent sections. The total volume of each lesion is the sum of the section lesion volumes in the series of sections, for each spinal cord.

Statistical analyses were carried out using SPSS and GraphPad Prism software. When equal variances could be assumed, the differences between groups were tested by unpaired, two-tailed Student’s t-test (for two groups) or by One-Way ANOVA followed by multiple comparisons Bonferroni post-hoc corrections (for more than two groups). Otherwise, unpaired, Mann-Whitney U-tests were performed for two groups.

## Results

### Prox1 is expressed in OPCs and OLs in primary cells and *in vivo*

**Fig 1:**
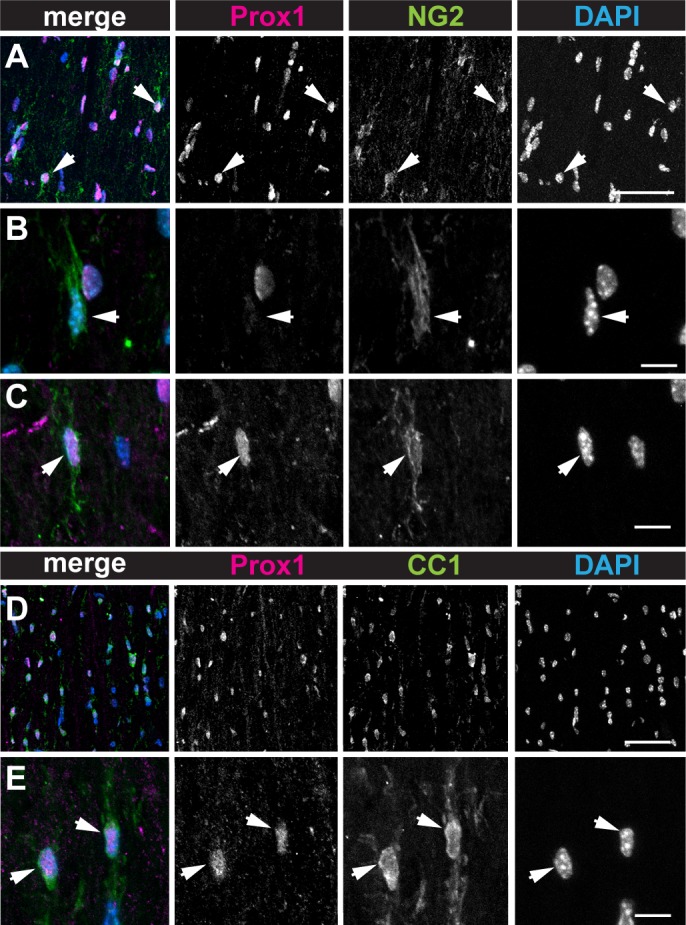
Prox1 is distributed in the OL cell lineage in the mouse spinal cord. (A-C) Prox1 is distributed in OL progenitor cells (OPC) as identified by colocalisation with NG2. (B,C) Higher magnification views showing that some NG2+ cells have little or no Prox1 signal (B, arrows), whereas others have high Prox1 signal (C, arrows). (D,E) Prox1 is distributed in OLs (OL) as identified by colocalisation with CC1. (E) Higher magnification views, showing CC1+ Prox1+ cells (arrowheads). Scale bars: (A,D) 50μm; (B,C,E) 10μm.

To test whether in the mouse *Prox1* might be expressed in the OL cell lineage, we examined the distribution of Prox1 protein in adult mouse spinal cords using anti-Prox1 antibodies and double immunostaining with anti-NG2 to identify OPCs and anti-CC1 (CC1) for OLs. Whereas most NG2-positive OPCs were Prox1-negative (Fig 1A and 1B), 31.9–48.6% of NG2+ OPCs with dendritic processes also stained with anti-Prox1 (n = 105 scored NG2+ cells at 4 weeks of age in one wild-type mouse; 48.6% is NG2+ Prox1+, and n = 150 NG2+ cells at 8 weeks in *Prox1-CKO+/-* heterozygous intact 3 mice (see below); average: 31.9% NG2+ Prox1+/NG2)(Fig1A and 1C). In contrast, virtually all CC1+ OLs in the white matter were also Prox1+ (Fig 1D and 1E) (n = 85 scored CC1+ cells; 94.1% CC1+ Prox1+/CC1+ in one wild-type mouse; and n>500 CC1+Prox1+ cells in *Prox1-CKO+/-* heterozygous intact 3 mice (see below), average: 93.6% Prox1+CC1+/CC1+). The difference in Prox1+ expression in OPCs suggests either that there are two types of OPCs (some NG2+ Prox1—and some NG2+ Prox1+) or that OPCs gradually increase Prox1 protein levels over time to result in all OLs expressing *Prox1 in vivo*. The invariable distribution in OLs suggests a prominent function for Prox1 in OLs.

**Fig 2:**
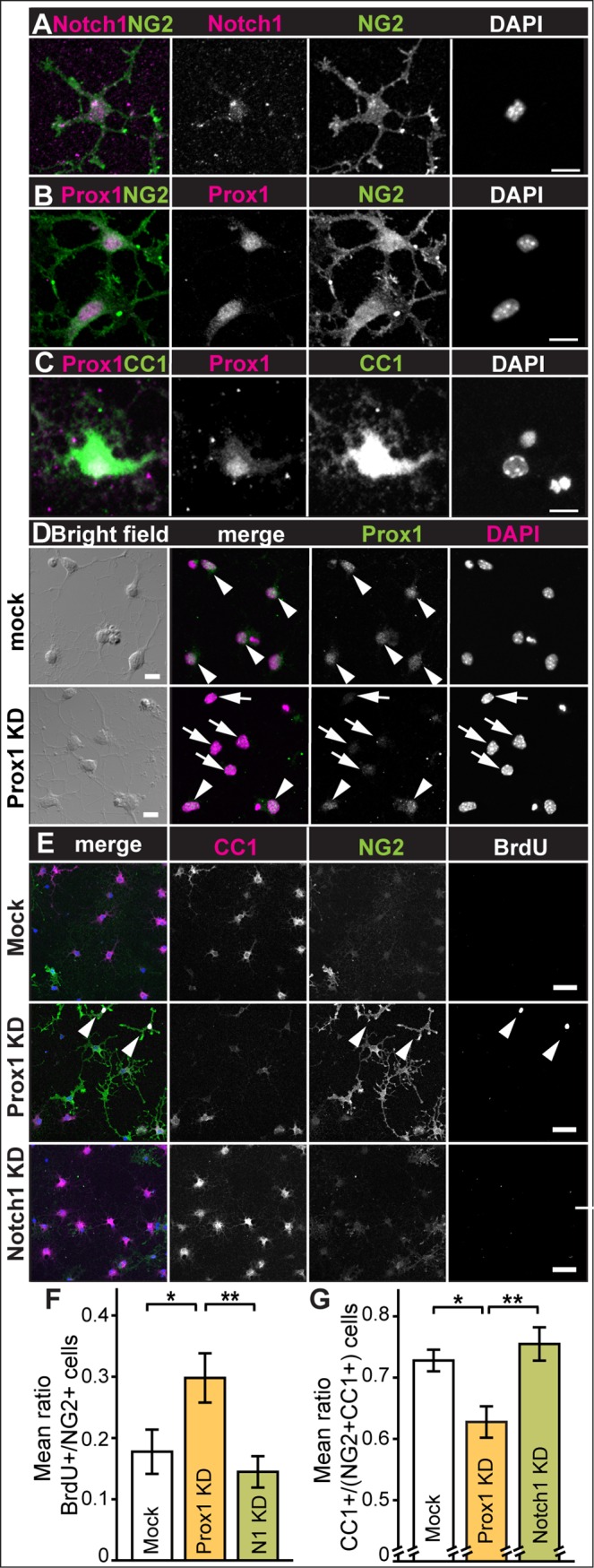
Prox1 inhibits proliferation and is required for differentiation of primary OPCs. Immunostaining of primary OPCs showing that NG2+ OPCs are (A) Notch1+, note that this anti-Notch1 antibody also detects nuclear Notch^ICD^ and (B) Prox1+. (C) Prox1 is present in CC1+ OLs. (D, E) Prox1 knockdown by siRNA reduces OL differentiation. (D) Control mock-transfected cells have Prox1, whereas in *prox1-siRNA* transfected cells some OPCs retain Prox1 (arrowheads), other cells have dramatically reduced Prox1 (arrows). (E-G) In differentiation medium, control mock-transfected primary OPCs became mostly CC1+ differentiated OLs, whereas transfection with *prox1-siRNA (Prox1KD)* increased the proportion of NG2+BrdU+ cells, and transfection with *Notch1-siRNA (Notch1KD)* increased the proportion of CC1+ cells (E). (F,G) Quantifications: (F) mean number of BrdU+ amongst NG2+ cells, in control cells (mock) and upon transfection of *Prox1-siRNA* (KD: knockdown) or *Notch1-siRNA* (N1 KD). (G) Mean ratio of CC1+ cells over the total cells (NG2+ CC1+) in control cells (mock) and cells transfected with *Prox1-siRNA* (KD: knockdown) or *Notch1-siRNA* (KD). Error bars represent standard error of the mean; asterisks: *p<0.05, **p<0.01. One Way ANOVA p<0.05, followed by Bonferroni post-hoc multiple comparison corrections. Sample sizes: mock: n = 9; *Prox1-siRNA*: n = 10; *Notch1-siRNA*: n = 9. Scale bars: (A-D) 10mm, (E) 50mm.

Primary OPCs in culture were NG2+, Notch1+ (Fig 2A), and remarkably, also Prox1+ (Fig 2B). Consistent with the *in vivo* results, virtually all CC1+ OLs differentiated from purified primary OPCs, were also Prox1+ (Fig 2C).

Altogether, these data indicate that Prox1 expression in OPCs varies and Prox1 is prominent and invariably distributed in differentiated OLs.

### Prox1 inhibits OPC proliferation and is required for OL differentiation in cell culture

To test what function might Prox1 have in the OL cell lineage, we asked whether Prox1 siRNA knock-down might affect proliferation or differentiation of mouse primary OPCs in culture. We transfected OPCs with *Prox1-siRNA* and *Notch1-siRNA*. In *Prox1-siRNA* transfected OPCs, Prox1 signal was either weakened or undetectable compared to mock transfection controls (Fig 2D, 71% Mock transfected OPCs are Prox1+ n = 31 scored cells vs. 16% of *Prox1-siRNA* transfected OPCs n = 43 scored cells). To test whether *Notch1-siRNA* or *Prox1-siRNA* affected OPC proliferation, the cell proliferation marker BrdU was applied. Whereas *Notch1-siRNA* had no effect, transfection of primary OPCs with *Prox1-siRNA* resulted in a significant increase in BrdU incorporation by NG2+ cells, compared to control (p<0.05) and to *Notch1* knockdown (p<0.01), indicating that Prox1 inhibits OPC proliferation (Fig 2E and 2F)(n = 100–200 cells scored for BrdU+, NG2+ and CC1+ per well, x 9–10 repeats). To test whether Notch1 or Prox1 knockdown affected OPC differentiation into OLs, we quantified the number of CC1+ OLs relative to total cell number [CC1+/(NG2+ and CC1+)] upon siRNA transfection. *Notch1-siRNA* had no effect, but transfection of primary OPCs with *Prox1-siRNA* resulted in a significant decrease in relative CC1+ OL cell number, compared to mock (p<0.05) and *Notch1* (p<0.01) transfections (Fig 2E and 2G), meaning that Prox1 is required for OL differentiation. Consistently with the role of Notch1 in inhibiting OL differentiation [14,29], our knockdown results suggested opposite functions for Prox1 and Notch 1 in the OL lineage. In fact, BrdU+ and CC1+ cell counts differed significantly between Prox1-knockdown and Notch1-knockdown, and to a greater extent than between Prox1-knockdown and mock transfection controls (Fig 2F and 2G).

Altogether these data from primary cells in culture show that Prox1 inhibits NG2+ OPC proliferation and is required for CC1+ OL differentiation.

### Conditional knockout reveals that Prox1 is required for OL differentiation *in vivo*

**Fig 3:**
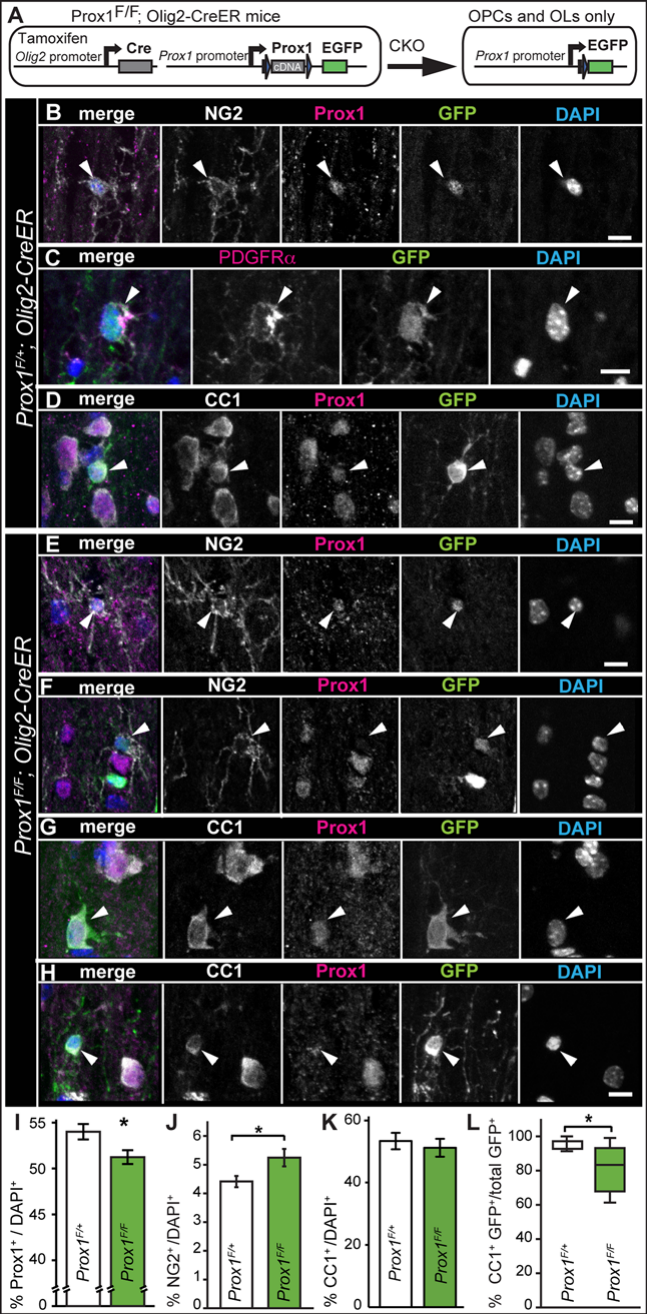
Prox1-CKO in Olig2+ cells increased the number of OPCs and reduced OL number. (A) Diagram illustrating that CKO in OL cell lineage results in the loss of Prox1 and the expression of GFP under the endogenous *Prox1* promoter. (B-D) *Prox1CKO+/-* knockout GFP+ heterozygous cells were generated in *Prox1F/+; Olig2-CreER* mice within the OL cell lineage. (B,C) GFP was found in NG2+ Prox1+ (arrowheads; B) and PDGFRα+ (arrowheads, C) OPCs. (D) GFP was found in CC1+ Prox1+ oligodencrocytes (arrowheads). (E-H) After 7 weeks of tamoxifen application, weak anti-Prox1 signal was still detectable in some GFP+ NG2+ OPCs (E, arrowheads), whilst others lacked any Prox1 signal (F, arrowheads). GFP+ CC1+ OLs either had Prox1 (G, arrowheads), or very weak to no Prox1 signal (H, arrowheads). (I-K) Quantification of Prox1+, NG2+ and CC1+ cells in the ventral funiculus of mouse spinal cords. (I) The number of Prox1+ cells relative to the total, decreased in homozoygous *Prox1-CKO-/-* mice. (J) The number of NG2+ OPCs relative to total cell count increased in homozygous *Prox1-CKO-/-* mice. (K) The number of CC1+ cells relative to the total does not change significantly in homozoygous *Prox1-CKO-/-* mice. (L) Box-plot showing that amongst the *Prox1-CKO-/-* cells identified as GFP+ there was a reduction in CC1+ OLs. * p<0.05 (I,J) Student t-test; (L) Mann-Whitney U-test; n = 6–7 mice. Error bar: standard error of the mean. (I-L) All are *Olig2-CreER*. (B-H) Scale bar, 10 μm.

To ask whether Prox1 might also regulate OPC proliferation and differentiation *in vivo*, we used Prox1 conditional knockout (*Prox1-CKO*) mice to delete the *Prox1* gene only in the OL lineage using the *Olig2-CreER* mouse line [23,24]. Upon Tamoxifen treatment, *GFP* is expressed under the control of the endogenous *Prox1* promoter. The mice of genotype *Prox1* ^*Flox/+;*^ *; Olig2-CreER* can generate GFP+ *Prox1-CKO+/-* heterozygous cells, and *Prox1* ^*Flox/Flox*^ *;Olig2-CreER* mice can generate GFP+ *Prox1-CKO-/-* homozygous mutant cells, within the OL lineage (Fig 3A). Consistently with the above data from in vivo and primary OPCs, in heterozygous *Prox1* ^*Flox/+;*^ *; Olig2-CreER* mice, few GFP+ cells were NG2+ or PDGFRα+ OPCs with dendritic morphology (Fig 3B and 3C), whilst most GFP+ cells were CC1+ OLs (Fig 3D). This confirms that Prox1 is expressed in OPCs but most prominently in differentiated OLs.

In *Prox1* ^*Flox/Flox;*^ *; Olig2-CreER* homozygous mice (i.e. *Prox1-CKO-/-*), GFP+NG2+ OPCs with dendritic morphology had weak or no Prox1 signal (Fig 3E and 3F), and CC1+ GFP+ OLs could be either Prox1+ (Fig 3G) or Prox1− (Fig 3H), suggesting that the penetrance of knock-out events was incomplete. Furthermore, not all *Prox1-CKO-/-* null mutant OPCs may be detectable with GFP. This is because Prox1 can regulate its own expression, thus *Prox1-CKO* can lead to loss of Prox1-promoter activity and result in GFP-negative cells [23]. Thus, to investigate the effects of *Prox1-CKO* we analysed the effects in total cell populations. Using anti-Prox1 antibodies, we found a significant 5% reduction of Prox1+ cells in the ventral funiculus of *Prox1-CKO-/-* homozygous mice compared to heterozygotes (Fig 3I, p<0.05).

We then asked how might *Prox1-CKO* affect NG2+ OPC and CC1+ OL cell number *in vivo*. Conditional knockout in *Prox1* ^*Flox/Flox;*^ *; Olig2-CreER* homozygous mice resulted in an increase in the relative number of NG2+ cells compared to heterozygous mice (Fig 3J, p<0.05). As we had shown above that primary OPCs proliferate more upon Prox1 knock-down, this suggests that *Prox1-CKO-/-* null OPCs proliferate more. In contrast, in *Prox1-CKO-/-* homozygous mice there was no significant difference in the number of CC1+ OLs over the total cell population (Fig 3K), but there was a significant reduction in the relative number of GFP+ CC1+ OLs (Fig 3L, p<0.05), suggesting that Prox1 is required for normal CC1 expression and OL differentiation. Altogether, Prox1-CKO in the OL cell lineage showed that Prox1 inhibits OPC proliferation and/or NG2 expression and it is required for OL differentiation and/or CC1 expression.

### Prox1 is required for OL differentiation upon LPC demyelination lesions in adult spinal cords

**Fig 4:**
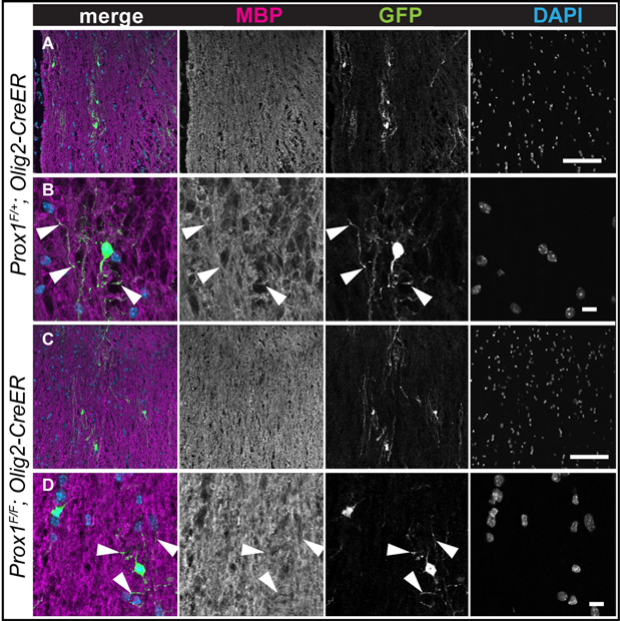
The overall MBP pattern is not affected in tamoxifen treated intact *Prox1 CKO* spinal cords. (A-D) Maximum projection of 2 confocal optical sections. Note that overlap of GFP+ processes and MBP staning is observed in both *Prox1-CKO+/-* and *Prox1 CKO-/-* (arrowheads); MBP staining is less pronounced in cell bodies, but its overall appearance is not distinguishable between the two genotypes. Scale bars: (A, C) 100 μm; (B, D) 10 μm.

Demyelinating lesions induce a glial regenerative response, that is, an increase in OPC proliferation, spontaneous OL differentiation and remyelination [30–33]. Thus, we wondered whether *Prox1-CKO* would affect this response. Lysolecithin (LPC) is frequently used to induce demyelination in rodents, and demyelination is assessed by loss anti-Myelin Basic Protein (MBP) in white matter [30,31,33]. To investigate the effect of *Prox1-CKO* in the glial regenerative response to LPC lesions, we first tested whether tamoxifen application to induce *Prox1-CKO* would in itself affect overall myelination and the MBP pattern in the spinal cord. Following tamoxifen application at 4–5 weeks, the overall MBP pattern in the spinal cord of treated mice did not change in *Prox-CKO -/-* mice compared to the heterozygous controls (Fig 4A and 4C). Furthermore, *Prox-CKO -/-* cells were identified as GFP+ and they had MBP protein in their projections (Fig 4B and 4D). Thus, tamoxifen treatment and *Prox1-CKO* did not in themselves affect the overall MBP pattern.

**Fig 5:**
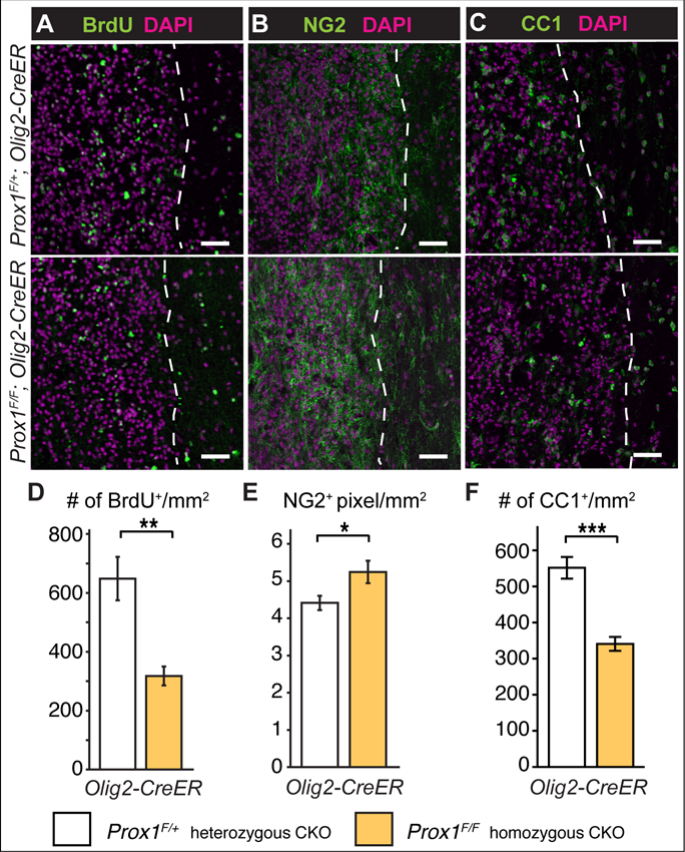
LPC induced demyelination lesions in Prox-CKO-/- mutant mice had fewer OLs and greater NG2+ area. (A-C) Immunostaining with (A) BrdU, (B) NG2 and (C) CC1, in LPC-induced lesions in the spinal cords of *Prox1-CKO+/-* heterozygous (top) and *Prox1-CKO-/-* homozygous (bottom) mice, quantification in (D-F). Note that lesions have a higher nuclear (DAPI) density than surrounding tissue. (A,D) Fewer BrdU+ cells were observed within the lesions of *Prox1-CKO-/-* mutant mice. *Prox1-CKO+/-* n = 5 and *Prox1-CKO-/-* n = 4. (B,E) *Prox1-CKO-/-* lesions had increased NG2+ signal area. *Prox1-CKO+/-* n = 9 *and Prox1-CKO-/-* n = 8. (C,F) There were fewer CC1+ OLs within the lesions of *Prox1-CKO-/-* mice than in controls. *Prox1-CKO+/-* n = 8 and Prox1-/- n = 6. *** p<0.001;** p<0.01; * p<0.05 Student t-test; error bar: standard error of the mean. Dashed lines indicate the lesion boundary. Scale bar, 50 μm.

LPC induced demyelination [32] was applied by injections into the spinal cord, in combination with BrdU incorporation (2 days post-LPC injection), in *Prox1-CKO* mice (Tamoxifen at 4–5 weeks, LPC injection at 8–9 weeks, perfusion at 14 days post-LPC injection). The largest MBP negative lesions had epicenters that were sparsely populated with NG2+ and CC1+ cells, consistently with reports that oligodendrogenesis progresses from the border of the lesions [6,7,34]. Thus, we focused on a 100 μm wide band inward from the lesion border, which was identified from the MBP-negative boundary and the boundary of high density DAPI+ nuclei accumulation. There were fewer BrdU+ cells in *Prox1-CKO-/-* homozygotes than in heterozygotes (Fig 5A and 5D, p<0.01)(see below). It was not possible to count the NG2+ cells, as the NG2 immunoreactivity formed a continuous mesh over the entire lesion (Fig 5B), and individual cells could no longer be reliably distinguished, thus we measured the area covered by NG2+ signal. The extent of NG2 distribution over the lesions was greater in *Prox1-CKO-/-* homozygotes than in heterozygotes (Fig 5B and 5E p<0.05). Although net BrdU+ cell number was reduced in *Prox1-CKO-/-* homozygotes, this suggests that in the absence of Prox1, LPC lesions induced either increased OPC proliferation or NG2 expression by OLs. There were also fewer CC1+ OLs in LPC-injected homozygous *Prox1-CKO-/-* mice than in the heterozygotes (Fig 5C and 5F, p<0.001), showing that LPC treatment is a sensitized condition that enhances the penetrance of the *Prox1* loss of function phenotype. This further supports the notion that Prox1 is required for OL differentiation.

**Fig 6:**
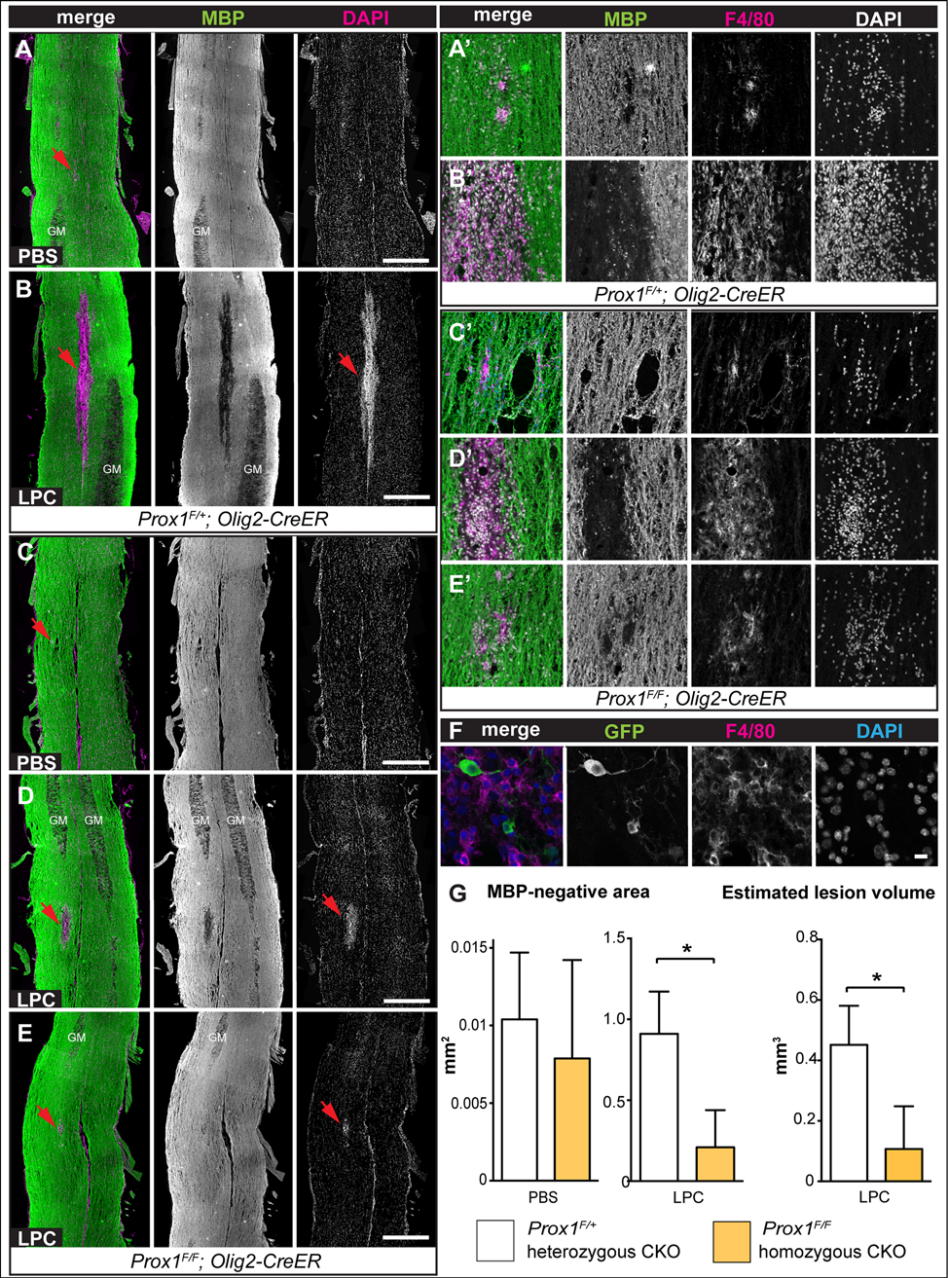
LPC induced demyelination in the spinal cord of Prox1-CKO mice resulted in smaller lesions. (A-E) LPC injection in the spinal cord induced demyelination lesions in the white matter visualised as anti-MBP-negative areas (arrowheads), which were smaller in *Prox1-CKO-/-* mice compared to heterozygous controls (compare B with D and E). In 2 out of 6 *Prox1-CKO-/-* mice, the MBP-negative lesions were extremely small (E). Nuclear staining with DAPI revealed high cell density within the MBP-negative areas. (A’-E’) High magnification details of the MBP-negative lesions indicated by arrowheads in (A-E), which accumulated F4/80+ macrophages. (F) GFP+ F4/80+ cells are not found in *Prox1-CKO+/-*, suggesting Prox1 does not directly influence macrophages in this context. (G) Bar graphs showing that upon PBS mock injection (left) MBP-negative lesions are comparable in size in control and *Prox1-CKO-/-* mice, but upon LPC injection (right) demyelinated MBP–areas are smaller in *Prox1-CKO-/-* mice. Total lesion volume (far right) was also reduced in *Prox1-CKO-/-* homozygotes compared to the heterozygous animals. Genotypes: Control: *Prox1F/+*, *Olig2-CreER* and *Prox1-CKO-/-*: *Prox1F/F*, *Olig2-CreER*. GM: gray matter; LPC: lysolecithin. * p<0.05, Student t-test. Sample sizes: control, n = 5; *Prox1-CKO-/-*, n = 6; Horizontal sections, rostral is up, scale bar: 500 μm.

Interestingly, conditional *Prox1-CKO* altered demyelination lesion size. Mock PBS injection did not induce demyelinated lesions in either *Prox1-CKO* heterozygous or homozygous mice (Fig 6A and 6C), despite the injection sites being reliably identified by the small accumulation of DAPI+ nuclei and F4/80+ activated macrophages or microglia (Fig 6A’ and 6C’). LPC injection in *Prox1-CKO+/-* heterozygous mice caused large MBP-negative lesions (Fig 6B). Consistently with previous studies, MBP− lesions were packed with DAPI+ nuclei and F4/80+ macrophages/microglia (Fig 6B and 6B’). In contrast, MBP− lesions were significantly smaller in *Prox1-CKO-/-* homozygous mice (Fig 6D, 6E and 6G) so that in extreme cases, the MBP− area was almost the same size as those in PBS-injection lesions, and with similar accumulations of DAPI+ and F4/80+ cells (Fig 6E and 6E’). Consistently, the estimated total lesion volume also significantly decreased in *Prox1 CKO-/-* homozygotes (Fig 6G, p<0.05; average estimated lesion volume: *Prox1-CKO+/-* 0.45 mm^3^ vs. *Prox1CKO-/-* 0.11mm^3^). These data suggested that *Prox1-CKO-/-* had a protective effect against LPC-induced injury.

The smaller lesions size and reduced accumulation of F4/80+ cells in *Prox1-CKO-/-* homozygous mutants might suggest that the net observed reduction in BrdU+ cells could have been due to a requirement for Prox1 in the proliferation of macrophages/microglia upon LPC induced lesions, resembling its function in OPCs. In fact, LPC treatment activates microglia that attack the myelin sheath, increasing lesion size [9,32,33,35]. However, *Olig2Cre* does not drive expression in microglia [36]. Furthermore, although there was a dramatic accumulation of F4/80+ active microglia/macrophages in LPC induced lesions in *Prox1-CKO+/-* heterozygotes, none of these cells co-distributed GFP with F4/80 (Fig 6F *Prox1-GFP*, n = 5), confirming that the *Olig2* promoter does not drive expression in microglia/macrophages, and revealing that Prox1 is not localised in these cell types under these experimental conditions. This means that Olig2-driven *Prox1-CKO* could not directly affect microglial/macrophage number, fate or migration. Alternatively, the reduction in microglia/macrophages could be an indirect effect of *Prox1* mutant OLs or OPCs (see discussion). Together, our data showed that upon LPC-induced demyelination, *Prox1-CKO-/-* in OL lineage cells led to the downregulation of CC1, upregulation of NG2, and prevented the formation of large demyelinated lesions.

## Discussion

**Fig 7:**
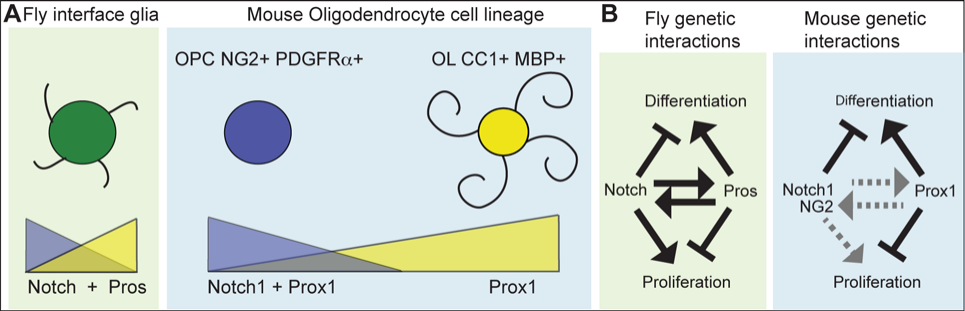
Prox1 function in the OL cell lineage. (A) Comparison of *Drosophila* enwrapping interface glia (IG) and mammalian OPCs and OLs. *Drosophila* glia are differentiated cells that enwrap axons, recycle neurotransmitters, proliferate upon injury and express both *Notch* and *pros*. OPCs are proliferative progenitors and have both Notch1 and Prox1. OLs are differentiated cells that myelinate axons, and have Prox1. (B) In *Drosophila* IG, Pros and Notch maintain each other’s expression but have opposite functions in cell proliferation and differentiation. This maintains these cells quiescent but ready to divide upon injury. In the mouse, hypothetical interactions between Prox1 and Notch1 are given: arrows in black indicate evidence presented here as data or based on OL cell lineage literature, and dotted in grey indicate hypothetical genetic interactions. Accumulation of Prox1 protein in OPCs could drive cell cycle exit, repress NG2 expression, and drive OL differentiation. Prox1 may be required in OLs to maintain differentiation and function.

Our data show that in the mammalian spinal cord, Prox1 inhibits OPC proliferation, is required for OL differentiation, and is involved in the glial regenerative response to demyelination injury (Fig 7).

We have shown that Prox1 is distributed in NG2+ and PDGFRα+ OPCs, and in CC1+ OLs in wild-type mouse spinal cords, in mouse primary cultured OPCs and in heterozygous *Prox1* ^*Flox/+;*^ *; Olig2-CreER* control mice *in vivo*. Whereas the distribution of Prox1 in NG2+ PDGFRα+ OPCs varied, virtually all CC1+ OLs had Prox1. Most likely, Prox1 is first expressed in OPCs, and protein levels increase over time, leading to OL differentiation. However, there is also cell type heterogeneity within the NG2+ cells, as not all of them in the white matter of the adult brain have mitotic potential, some behave as neural stem cells, and they can also differ in physiology [37]. Thus, perhaps Prox1 may be another indicator for two types of OPC populations. Prox1 expression has also been reported in oligodendrocyte lineage cells of the brain cortex [20]. Thus, together with our data, this indicates that Prox1 is present in the oligodendrocyte cell lineage throughout the mammalian CNS. Importantly, our data show that Prox1 is characteristic of differentiated OLs.

Our data showed that Prox1 inhibits OPC proliferation and is required for OL differentiation. Loss of Prox1 function increased OPC proliferation in primary OPCs, as the number of NG2+ OPCs with incorporated BrdU increased upon Prox1 siRNA knock-down compared to controls. NG2+ OPC cell number also increased in *Prox1-CKO-/-* in mice *in vivo*. The increase in OPC number upon loss of Prox1 *in vivo* is most likely due to induced OPC over-proliferation and failed OL differentiation. In fact, we have shown that Prox1 is required for OL differentiation. Loss of *Prox1* function led to fewer OLs both upon siRNA *Prox1* knockdown in primary OPCs, and in *Prox1-CKO* in the OL cell lineage in mice *in vivo*. The expression and loss of function analyses showed that Prox1 is present in all OLs and is required for their maintenance and differentiation.

Conceivably, Prox1 could regulate the above events by antagonizing Notch1 function in the OL cell lineage (Fig 6A and 6B). Like others [13,14], we also found Notch1 in mammalian primary OPCs in cell culture and *in vivo*, and that over-expression or knockdown of Notch1 in primary OPC cell culture had no effect. Remarkably, however, knockdown of Prox1 and Notch1 had opposite effects on proliferating OPCs *versus* OLs, and the difference between their effects was greater than the difference between Prox1-knockdown and the control. In *Drosophila* Interface glia, Pros and Notch antagonize each other’s function in cell proliferation and differentiation, whilst also maintaining each other’s expression [16,17]. In this way, as their expression is maintained through positive feedback, their antagonistic functions provide negative feedback, creating a homeostatic control of glial cell number. Functional relationships between Prox1 and Notch1 might take place in the mouse OL lineage, but they are unlikely to be identically conserved (Fig 7A and 7B). In *Drosophila* there is no difference between glial progenitors and differentiated glia. Instead, differentiated interface glia enwrap the neuropile and axons, proliferate upon injury, and have both Notch and Pros. In mammals, OPCs and OLs are distinct cell states, and whereas OPCs can have both Notch1 and Prox1, OLs have only Prox1. The functional relationship between Notch1 and Prox1 in the mammalian OL lineage could regulate the transition from dividing OPCs to differentiating OLs, as Prox1 levels rise to drive cell cycle exit and differentiation.

Although we observed increased proliferation when Prox1 was knocked-down in cultured OPCs, fewer BrdU+ cells were detected within the lesions of *Prox1-CKO* mice, than in controls. The overall reduction in BrdU+ cells could correspond to decreased proliferation of macrophages/microglia in *Prox1-CKO* mice, consistently with the smaller nuclei-dense areas upon LPC injection in the homozygous mutants. Nevertheless, OPCs may have proliferated less in *Prox1-CKO -/-* at a time of BrdU treatments, which can be explained in two ways. First, in *Drosophila*, *pros* mutant glia do not divide more times, but divide faster skipping the G1 phase [16,17]. Similarly, when we applied BrdU, OPCs may have already gone through S-phase, leading to an under-representation of the extent of cell division. Alternatively, genetic interactions involving a proliferation activator and Prox1 may be involved. In *Drosophila*, although Pros inhibits glial proliferation, Pros is required for glial proliferation to take place [16,17]. Whilst injury in the *Drosophila* CNS normally provokes glial proliferation, in *pros* mutants there is a relative reduction in the number of glial cells after injury instead [16,17]. This is because in the absence of Pros, Notch signaling also decreases. Furthermore, whilst NFκB triggers cell proliferation upon injury, its expression also depends on Pros [17]. Thus, in *pros* mutants, there is a reduction in both NFκB and Notch levels, and consequently glial cells cannot proliferate in response to injury [17]. In mouse brains, only half of OPCs are proliferative [38,39] and coincidently only half of OPCs expressed Prox1 in spinal cords, whilst in primary cultures all OPCs were potentially proliferative and expressed Prox1. Hence, like in fruit-flies and possibly in human brain tumours [21,22], Prox1 might confer mammalian glia with proliferative potential. If Prox1 –most likely indirectly—were to activate the expression of proliferation activators, then loss of Prox1 could lead to an initial increase in proliferation followed by a loss of mitotic potential, preventing injury-induced regenerative proliferation (Fig 7B).

Our data also revealed statistically significant differences in LPC lesion size between *Prox1-CKO-/-* homozygous and heterozygous mice. NG2 protein levels increased and spread over a larger area in demyelinated *Prox1-CKO-/-* mice than in controls. It is known that increased NG2 leads to increased OPC proliferation [40,41]. Thus, conceivably, in *Prox1-CKO* undetected increased OPC proliferation may correlate with smaller lesion size. Alternatively, the smaller lesion size in *Prox1-CKO-/-* mice could also be due to decreased lesion expansion. LPC lesions are caused by dissolution of membrane lipids, and myelin breakdown by activated T-cells, microglia and macrophages, which invade the lesions, attack myelin, inhibit OPC proliferation, NG2 function and axonal growth, causing lesion expansion and preventing regeneration and repair [9,30,32,33,35,42–49]. Similarly to our findings, NG2 knockout mice also have smaller lesions compared to controls, and this was caused by reduced infiltration of microglia [41]. NG2 is expressed in microglia and is required for their migration, hence resulting in reduced microglia invasion in the mutant [41]. In *Prox1-CKO-/-* mice the lesions also had fewer F4/80 cells, thus perhaps secondary damage and lesion expansion did not take place either. In lesioned *Prox1-CKO-/-* mice, invasion by microglia and macrophages might have been mitigated by their reduced proliferation, or reduced T-cell activation. These would require both *Olig2* and *Prox1* to be normally expressed in microglia/macrophages–which we found not to be the case–and in T-cells, which is unknown. Whilst this indicates that Prox1 may not directly affect microglia/macrophages, it could still affect them indirectly. In a similar scenario, loss of Cgt—which is highly expressed in OLs, and is required for the major component of myelin sheath galactocerebrosides—in knock-out mutant mice, causes a defect in nerve conduction as well as defective postnatal lymphopoiesis [50]. It has been shown that the nervous system, most particularly glial cells, controls hematopoietic stem and progenitor cells exit from bone marrow niches [50,51]. Therefore, conceivably *Prox1-CKO* in the OL lineage may indirectly influence immune cells. In any case, the most remarkable finding was that in *Prox1-CKO-/-* mice the smaller lesions had higher and broader NG2+ coverage. NG2 can protect against the destructive effects of macrophages [52]. Thus, our data suggest that in spinal cord demyelination, the down-regulation of Prox1 in the OL lineage causes the up-regulation of NG2, which is protective.

To conclude, the data show that Prox1 regulates proliferation and differentiation of the OL cell lineage, and is a relevant component of the endogenous regenerative response to CNS injury. The relationship between Prox1 and NG2 in CNS repair warrants further attention.
